# Individual synaptic vesicles mediate stimulated exocytosis from cochlear inner hair cells

**DOI:** 10.1073/pnas.1811814115

**Published:** 2018-11-21

**Authors:** Chad Paul Grabner, Tobias Moser

**Affiliations:** ^a^Synaptic Nanophysiology Group, Max Planck Institute for Biophysical Chemistry, 37077 Göttingen, Germany;; ^b^Institute for Auditory Neuroscience and InnerEarLab, University Medical Center Göttingen, 37075 Göttingen, Germany;; ^c^Auditory Neuroscience Group, Max Planck Institute for Experimental Medicine, 37075 Göttingen, Germany

**Keywords:** ribbon synapse, synaptic efficiency, multivesicular release, univesicular release, membrane capacitance

## Abstract

Spontaneous excitatory postsynaptic currents (sEPSCs) measured from the first synapse in the mammalian auditory pathway reach a large mean amplitude with a high level of variance (CV between 0.3 and 1). This has led some to propose that each inner hair cell (IHC) ribbon-type active zone (AZ), on average, releases ∼6 synaptic vesicles (SVs) per sEPSC in a coordinated manner. If true, then the predicted change in membrane capacitance (C_m_) for such multivesicular fusion events would equate to ∼300 attofarads (aF). Here, we performed cell-attached C_m_ measurements to directly examine the size of fusion events at the basolateral membrane of IHCs where the AZs are located. The frequency of events depended on the membrane potential and the expression of Ca_v_1.3, the principal Ca^2+^-channel type of IHCs. Fusion events averaged 40 aF, which equates to a normal-sized SV with an estimated diameter of 37 nm. The calculated SV volumes showed a high degree of variance (CV > 0.6). These results indicate that SVs fused individually with the plasma membrane during spontaneous and evoked release and SV volume may contribute more variability in EPSC amplitude than previously assumed.

## Significance

Synaptic transmission is codetermined by presynaptic and postsynaptic neurons. Therefore, to understand how the inner hair cell (IHC) signals to spiral ganglion neurons at the first synapse in the auditory pathway, here we directly studied individual membrane fusion events by making cell-attached membrane capacitance recordings from IHCs, for which the quantal size is debated. The observed fusion steps in membrane capacitance are consistent with the quantal hypothesis of synaptic transmission in which individual synaptic vesicles undergo exocytosis independently from each other. This finding, in conjunction with previous work, raises the exciting possibility that action potential generation can be triggered by the release of a single vesicle at the IHC synapse.

Sound information is transmitted from inner hair cells (IHCs) in the cochlea to the brain via spiral ganglion neurons (SGNs). These bipolar neurons receive excitatory input from IHCs and signal this information directly to the auditory brainstem as discrete action potentials (APs). Several studies have documented that the rate of spontaneous excitatory postsynaptic currents (sEPSCs) and evoked EPSCs (eEPSCs) depends on Ca^2+^-stimulated release of glutamate from IHCs (1–4). Modeling and experimental work indicates that the majority of these EPSCs can trigger an SGN AP (5). What remains less clear is how many synaptic vesicles (SVs) give rise to s/eEPSC and, so, to an AP.

The standard approach used to estimate the postsynaptic response to a single SV release event entails measuring postsynaptic currents (PSCs) under conditions of low release probability, e.g., by blocking presynaptic APs with tetrodotoxin. These miniature PSCs (mPSCs) are typically equated to the independent fusion of SVs. mPSC amplitude is widely reported to be insensitive to changes in extracellular Ca^2+^, and this holds true even when the frequency of spontaneous mPSCs is steeply enhanced by elevating extracellular Ca^2+^ levels (6, 7). In contrast, a PSC evoked by an AP often increases in amplitude as extracellular Ca^2+^ levels are elevated, which is widely interpreted as multiple SVs fusing in unison to create the evoked PSC (ref. 7; for review, see ref. 8). Whether this is multivesicular release per synapse (MVR, i.e., more than 1 SV release per AP) or recruitment of more synapses that support univesicular release (UVR) depends on the system at hand.

While not driven by APs, mammalian IHCs follow this general description in that (*i*) the eEPSC at the onset of a presynaptic depolarization is often larger than the sEPSCs and (*ii*) the mean sEPSC size is relatively constant during changes in event frequency and changes in extracellular Ca^2+^ levels (9, 10). Since each afferent SGN is thought to contact one ribbon-type AZ, where ∼10–30 SVs are thought to be in fusion competent states (11, 12), multiple SVs can be released per AZ even upon brief presynaptic depolarizations. However, there is controversy surrounding whether sEPSCs and the eEPSC observed during sustained presynaptic stimulation result from the fusion of a single SV (UVR) or from coordinated multivesicular release (cMVR). The original description concluded that cMVR of 4–8 SVs mediates IHC release events (3). A more recent study, derived from postsynaptic SGN recordings and mathematical modeling of the IHC ribbon synapse, concluded that UVR might govern IHC release events (9). Clearly, further work is required to elucidate the nature of exocytosis from mammalian IHCs.

Here, we made cell-attached membrane capacitance (C_m_) recordings of exocytosis from mouse IHCs, thereby avoiding many of the complications associated with the interpretation of postsynaptic recordings. Cell-attached C_m_ recordings have been made from various secretory cells and proven useful in resolving a wide range of vesicle sizes, from single SVs (13) to secretory granules (14), and within the same recording (15). This approach has also been utilized to distinguish between UVR and a form of MVR called compound exocytosis (13, 16, 17). Here, we take an approach that involves stimulating the membrane patch directly and show that SV fusion in IHCs is voltage-dependent and requires Ca^2+^ entry through Ca_v_1.3 channels. The stimulated fusion events have an equivalent SV diameter of 37 nm, close to that measured with electron microscopy, and the shape of the size distribution follows that expected for UVR. The estimated variance in SV volume was large (CV > 0.6). The findings suggest that mammalian IHC are not prone to cMVR during spontaneous and sustained exocytosis as suggested (3, 16), but rather release may be dominated by a UVR scheme. In essence, this may create a uniquely sensitive, highly efficient synaptic transfer of 1 SV for 1 AP. This signaling regime will protect the AZ from fatigue and allow high frequency signaling.

## Results

### Step-Like Full-Fusion Events Predominate.

For cell-attached C_m_ recordings, we targeted the pipette to the basolateral membrane at the modiolar face of IHCs in the apical turn of the cochlea taken from hearing mice (4–8 wk old). The basolateral portion of the IHC has the highest density of ribbons, as this is where the SGNs contact the IHC (18). The SGN dendrites were dislodged from the IHC with positive pipette pressure to free the presynaptic membrane. The thick wall patch pipettes used here had a series resistance of ∼2.5 MΩ, which is expected to have a 5 µm^2^ area of free membrane at the dome of the patch (13, 19). Given the large number of synapses (∼12 synapses per IHC at the tonotopic position of recording; ref. 18) with a mean nearest neighbor distance of 2 µm (18), we estimated that the large recording patches made from the basolateral pole of the IHC would have a fair chance of capturing an AZ. Finally, to achieve an optimal C_m_ signal-to-noise ratio, we used the hardware-based Lock-in amplifier method (20), and applied sinewave settings similar to those employed to monitor small vesicles ∼45 nm in diameter (13, 15).

**Fig. 1.**
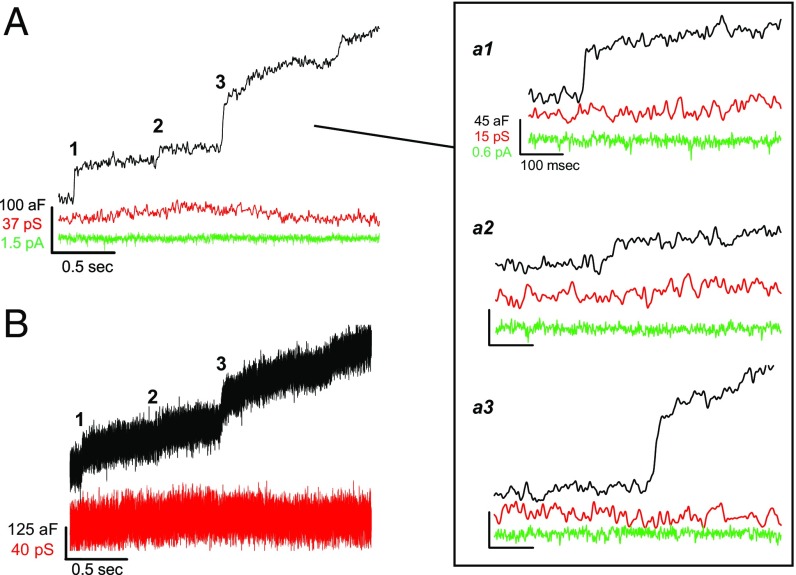
Full-fusion events are the most common. (*A*) Recording from a wild-type IHC that shows a staircase of up steps in C_m_ (black trace), which appear to lack correlated changes in conductance (red trace) or membrane current (green trace). (*a1*–*a3*) Magnified view of fusion events from *A* (individual steps *a1*–*a3* presented at same scale as in *a1*). (*B*) Same traces as in *A* (C_m_ and G), but not smoothed to reveal potential transient G signals associated with C_m_ fusion events. Lock-in sinewave settings: frequency, 58.5 kHz; *V*_rms_, 200 mV.

The exemplar traces presented in Fig. 1*A* show a series of step-like jumps in C_m_ measured from a wild-type IHC. Neither the corresponding conductance nor membrane current traces showed transitions that correlated with the steps in C_m_. This indicates the Lock-in signals were well separated from one another (further described in *SI Appendix*, Figs. S1 and S2). These simple up steps in C_m_ without subsequent down steps are referred to as “full-fusion” events (13). As reported here for IHCs, and in many other preparations (13–15, 21), full fusion was the most common type of event. Of nine cells, six exhibited only full-fusion events, and from the remaining three cells, 92% of the events were full fusion (185/201 events, from three patches). The other 8% of the fusion events were followed by a decline in C_m_ that was slower than the preceding C_m_ up step (rising and falling phase time constants: 3.3 ± 0.7 ms and 66.3 ± 18.2 ms, respectively*; P* = 0.002, *n* = 16) (*SI Appendix*, Fig. S3 *A*–*D*). These infrequent, peculiar events likely resembled “pseudo-flicker” fusion (22), and they differ from transient fusion events that are often called “kiss-and-run” and characterized as having a fast rise in C_m_ and a similarly fast fall in C_m_ (21, 23). In some situations, such as pituitary lactotrophs, a high percentage of the fusion events are kiss-and-run (24), and certain perturbations can increase the frequency of kiss-and-run events (24, 25).

The Real component of the Lock-in signal is an aggregate of conductances. In some instances, changes in membrane current (*I*_m_) were clearly reflected in the G trace, which we designate as changes in membrane conductance (*G*_m_) (*SI Appendix*, Fig. S1). Another signal potentially carried in the G trace can be related to a fusion pore conductance (*G*_pore_), which arises when a resistive pore forms between the vesicle and plasma membrane (for review, ref. 26). After inspection of traces with and without smoothing (as in Fig. 1 *A* and *B*), a *G*_pore_ signal could not be found (*SI Appendix*, *Signal Processing* and Fig. S2). Other studies have reported that small vesicle full-fusion events are not (15), or rarely accompanied by a *G*_pore_ signal (1.4% of events; ref. 13). Since the *G*_pore_ signal is directly proportional to vesicle size (27, 28), it has been studied extensively for large granules that are 200–1,000 nm in diameter and yielded large fusion events (C_m_ steps > 1 fF) (for review, ref. 26). In contrast, IHCs have the smallest SVs (∼36 nm) to be evaluated to date with the on-cell C_m_ method; thus, the *G*_pore_ signal may be too small to detect as a previous analysis predicted (29).

### Fusion Is Dependent on Voltage-Dependent Calcium Channels.

**Fig. 2.**
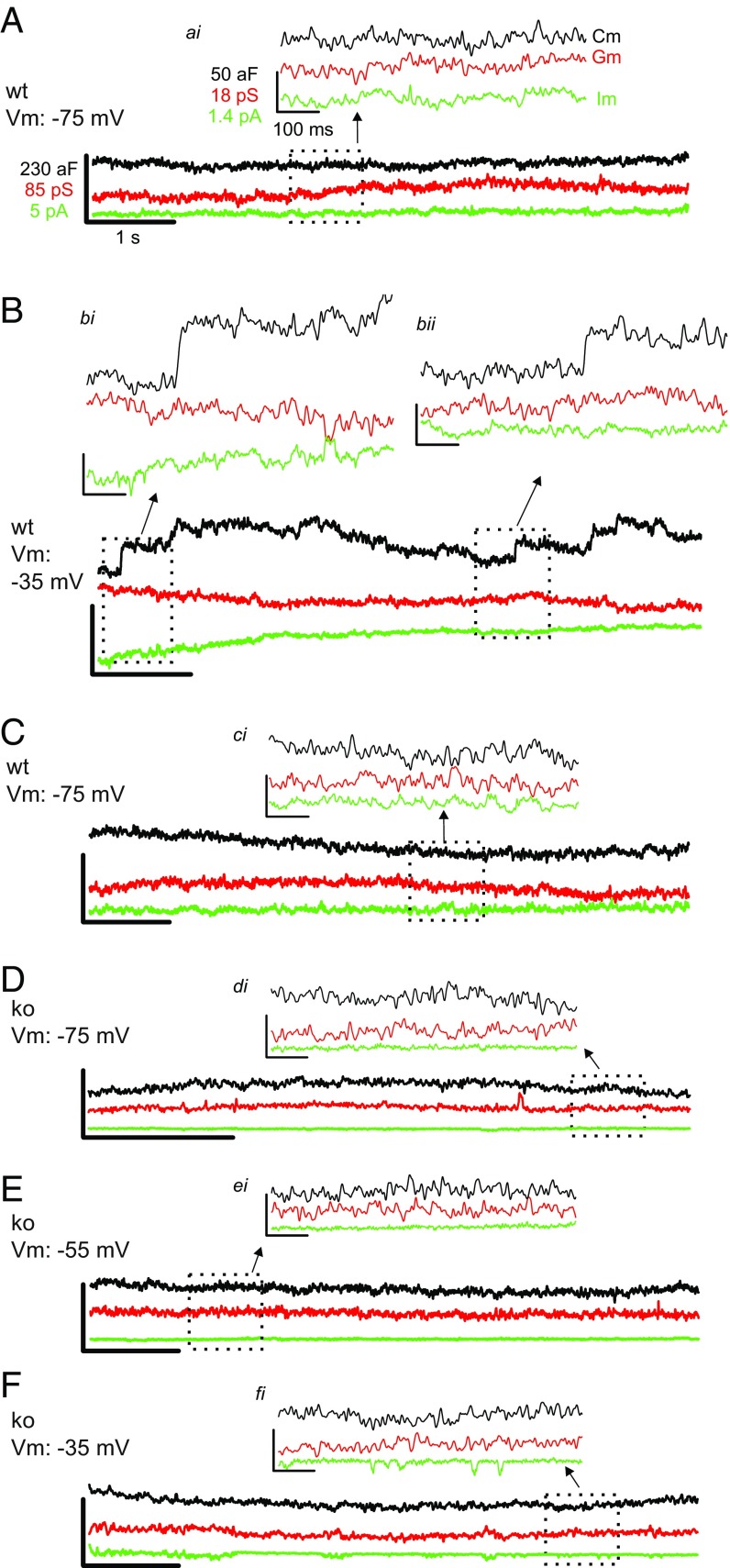
Example responses measured from wild-type (wt) and Ca_v_1.3-ko IHCs given depolarizing steps. (*A*–*C*) Chronology of on-cell recordings made from a wild-type cell with the patch *V*_m_ varied from −75 mV (*A*) to −35 mV (*B*), and back to −75 mV (*C*). (*Insets ai* and *ci*) Zoomed views illustrate a lack of fusion events at *V*_m_ = −75 mV. (*B*) Patch at *V*_m_ = −35 mV exhibits fusion events observed as upward steps in C_m_ without mirrored changes in membrane conductance (*G*_m_) or membrane current (*I*_m_). Two example fusion events are presented in *bi* and *bii*. (*D*–*F*) On-cell recordings made from an individual Ca_v_1.3-ko IHC patch held at *V*_m_ = −75 mV (*D*); *V*_m_ = −55 mV (*E*); *V*_m_ = −35 mV (*F*). Traces have the same scale bar values as in *A*, and the *Insets* are scaled to the values as shown in *ai*. Lock-in sinewave settings: frequency, 58.5 kHz; *V*_rms_, 150 mV.

**Fig. 3.**
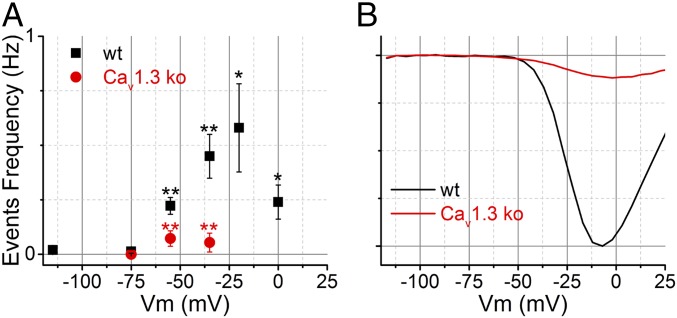
Summary of fusion event frequency for wild-type (wt) and Ca_v_1.3-ko IHCs. (*A*) Plot of event frequency for wild-type (*n* = 8 at rest, and *n* = 7 depolarized) and Ca_v_1.3-ko IHCs at different *V*_m_ (*n* = 5). (*B*) Normalized *I*_Ca_ for wild-type and Ca_v_1.3-ko IHCs taken from Brandt et al. (32). Black asterisks indicate statistical comparison between wild type at rest (*V*_m_: −75 mV) versus at depolarized *V*_m_ values, and red asterisks compare wild type and ko at the same *V*_m_ (*P < 0.05 and **P < 0.01).

Previously, researchers elevated extracellular KCl to depolarize the cell and stimulate vesicle fusion, because direct voltage stimulation of patches via the patch pipette rarely evoked fusion (13–15, 21). In contrast, we find that the event frequency in membrane patches of wild-type IHCs is sensitive to voltage changes applied to the patch pipette relative to the ground electrode. At the resting membrane voltage (*V*_rest_), the event frequency was 0.22 ± 0.04 Hz (*n* = 8 cells) (*V*_rest_ assumed to be −55 mV; ref. 30). When the patches were hyperpolarized by 20 mV (to *V*_m_: −75 mV, Fig. 2 *A* and *C*), there was a consistent suppression of event frequency (0.01 ± 0.01 Hz; 22-fold decrease from *V*_rest_ with *P* = 0.001; *n* = 5). When the patches were depolarized by 20 mV (to *V*_m_: −35 mV, Fig. 2*B*), the event frequency increased by twofold above *V*_rest_ (0.45 ± 0.1 Hz; significant increase from rest, *P* = 0.044; *n* = 7). The dependence of event frequency on *V*_m_ is summarized in Fig. 3*A*. The stimulated event frequency reported here is similar to what others reported when using KCl (13, 15) or other secretagogues (31) to initiate fusion in preparations studied with the on-cell configuration.

Given that the loss of Ca_v_1.3 from IHCs [via disruption of the Ca_V_1.3α_1_ gene: Ca_v_1.3 knockout (ko)] leads to >90% reduction in whole-cell calcium current (4) (see also Fig. 3*B*), and a dramatic impairment in exocytosis as judged by whole-cell C_m_ recordings (32), IHCs from Ca_v_1.3 ko mice were studied to address whether Ca_v_1.3 supports stimulated SV fusion in the on-cell configuration. Fig. 2 *D*–*F* shows recordings from a Ca_v_1.3 ko IHC that was silent at *V*_rest_ and when depolarized. On average, the frequency of fusion was not significantly altered when patches from Ca_v_1.3 ko IHCs were held at rest *V*_m_: −55 mV (0.07 ± 0.04 Hz; *n* = 5) versus depolarized to *V*_m_: −35 mV (0.05 ± 0.04 Hz; *P* = 0.75, *n* = 4). The event frequencies measured from wild-type IHCs held at *V*_m_: −55 mV and −35 mV were 4- and 11-fold higher, respectively, than what was observed for Ca_v_1.3 ko IHCs held at the same *V*_m_ values (sig. diff. *P* < 0.008; Fig. 3*A*). The results suggest that coupling of the voltage stimulus to SV fusion is mediated by Ca_v_1.3 in the on-cell configuration, lending further support to the specificity of the C_m_ events as reporting Ca^2+^-triggered SV fusion.

### Fusion Event Amplitude Follows a Normal Vesicle Size Distribution.

**Fig. 4.**
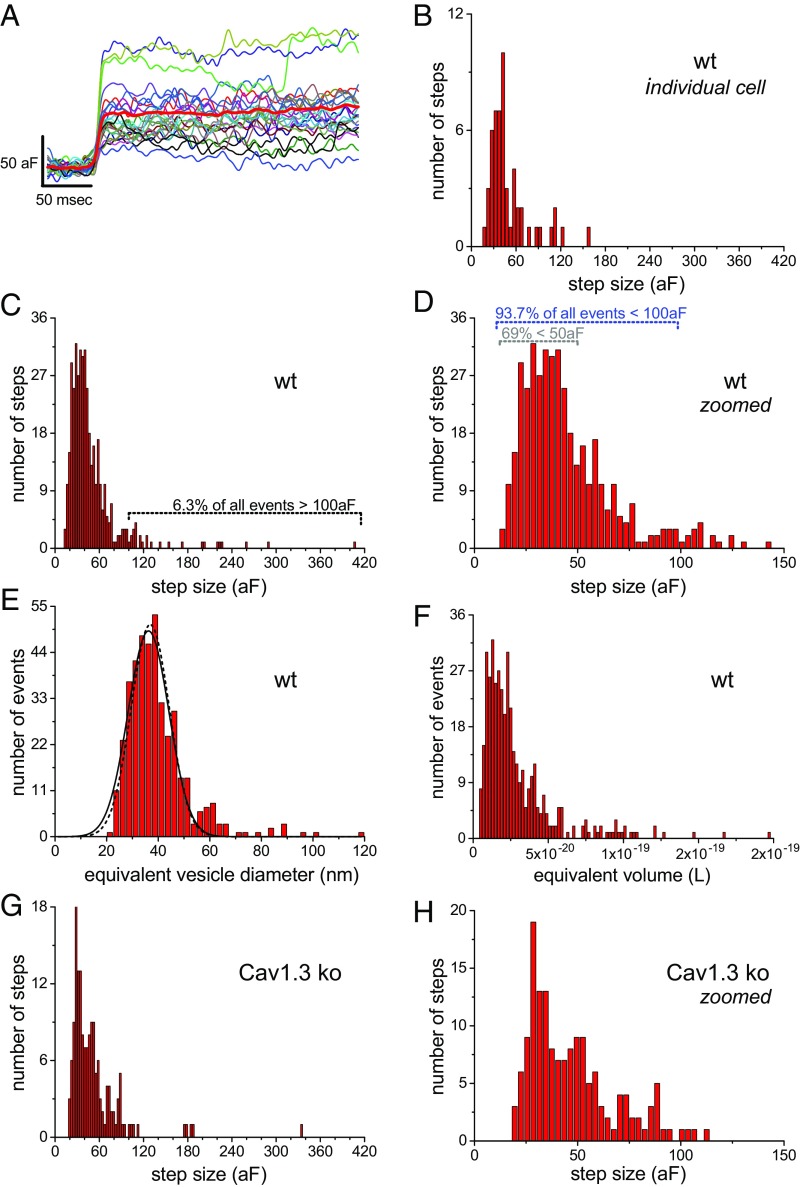
Fusion event amplitudes fall within the range of SVs common to IHCs. (*A*) Fusion events from a wild-type (wt) cell are aligned to their rising phase (average: thick red line). (*B*) Fusion event amplitude distribution for the patch in *A*. (*C*) Full range of wild-type event amplitudes. (*D*) Zoomed view of *C*. (*E*) Transformation of wild-type event amplitudes into full range of equivalent vesicle diameters (solid line is the Gaussian fit to equivalent diameter data; dashed line is SV diameter from EM; ref. 33). (*F*) Plots the full range of calculated vesicle volumes. (*G*) Full range of fusion event amplitudes recorded from Cav1.3 ko IHCs. (*H*) Zoomed view of *G*.

To estimate the amplitude of fusion events, patches with low noise levels were analyzed [∼4 attofarads (aF) rms noise]. An overlay of fusion events measured from an individual wild-type IHC highlights the variability in step height (Fig. 4*A*; only one patch recorded per cell). The distribution of event amplitudes measured from this cell peaked around 40 aF and was skewed toward larger sizes (Fig. 4*B*). Likewise, the plot of 416 events acquired from seven wild-type cells peaked around 40 aF and skewed rightward (Fig. 4 *C* and *D*). Only 1.9% (8/416) of the events were >200 aF, 6.3% (26/416 events) were >100 aF, and the majority of events, 69% (285/416), were <50 aF (Fig. 4*C*). The average fusion event was 40.2 ± 3.3 aF (mean ± SE; *n* = 7 patches), and this was made by averaging the median step amplitude derived from each cell (on average 59 events per patch; range: 29–99 events per patch). By using a specific capacitance of 9.1 fF/µm^2^ and assuming a spherical vesicle geometry (21), the equivalent SV diameters were calculated for the different size ranges. Only 1.9% of the events are estimated to be >83.6 nm (200 aF), 6.3% are >59.1 nm (100 aF), the majority (69%) are <41.8 nm (50 aF) (Fig. 4*E*), and the mean diameter calculated from the median per cell was 37.3 ± 1.1 nm (mean ± SE). Making a grand average over 93.7% of all equivalent vesicle diameters, those <59.1 nm, gave a value of 37.3 ± 7.8 nm (mean ± SD; 390 events, *n* = 7 cells), and a coefficient of variation (SD/mean) = 0.21. The resulting plot of diameters when fit with a Gaussian function (solid line, Fig. 4*E*) gave a distribution centered at 36.2 nm and a SD = 9.3 nm (CV = 0.26), which is in good agreement with the grand average, and indicates that the event amplitudes are normally distributed. These estimates closely approximate electron microscopy results that report normally distributed SV diameters with a mean value of 36.9 nm and a CV = 0.20 (33) (dashed line in Fig. 4*E*). However, conversion of C_m_ step values into equivalent SV volumes, which should be proportional to transmitter content of a SV (34–36), yields a distribution that is highly skewed (Fig. 4*F*), with a CV = 1.57 when calculated from all events (5.53 × 10^−20^/3.52 × 10^−20^ L). Using steps <100 aF gives a CV = 0.65 (1.61 × 10^−20^/2.48 × 10^−20^ L). Published CVs for EPSC measured from mammalian IHCs range from 0.3 to 1 (3, 9, 37, 38), and the estimated variance in SV volume falls within this range.

### Stimulation Conditions Did Not Alter the Event Amplitude Distribution.

It is known that stimulation conditions (9, 16, 25, 39) and genetic manipulations (16, 40) can influence quantal release properties (for review, see ref. 41). Therefore, we first examine whether fusion event amplitude is influenced by the expression of Ca_v_1.3 channels, a manipulation that greatly impacted the frequency of events (Fig. 3*A*). The plot of event amplitude for Ca_v_1.3 ko (Fig. 4 *G* and *H*) appears similar to that of wild type (Fig. 4 *C* and *D*). Only <1% (1/160) of the events were >200 aF, 6% (9/160 events) were >100 aF, and the majority of events, 62% (99/160), were <50 aF (Fig. 4*C*). The median step size calculated per patch gave a value of 41.3 ± 3.9 aF (mean ± SE; *n* = 5 patches). When comparing the average median amplitudes, no significant difference between the two groups was observed (*P* = 0.83; Table 1). Next, event amplitude was compared for the resting (*V*_m_ ∼ −55 mV) and depolarized conditions (*V*_m_ values −35 to 0 mV). As described above, the wild-type IHCs showed an increase in event frequency when depolarized from resting *V*_m_, but this had no bearing on their fusion event amplitude (wild-type, rest vs. depolarized: 43.0 ± 4.0 vs. 38.5 ± 3.6 aF; *P* = 0.42; seven patches; Table 1). In similar fashion, the C_m_ steps in Ca_v_1.3 ko IHCs had the same amplitude at *V*_rest_ and when depolarized (Table 1).

**Table 1.** Average fusion event properties

	Step amplitude, aF	
Ca_v_1.3 genotype	Wild type	Ko
Rest	43.0 ± 4.0	40.4 ± 2.9
Depolarized	38.5 ± 3.6	36.0 ± 6.2
Rest + Depolarized	40.2 ± 3.3	41.3 ± 3.9

Values are presented as mean ± SE and calculated from the median step size per stimulation condition, across five knockout (ko) and seven wild-type patches. Two-sample *t* test comparisons made between stimulation conditions on the same background, and between genetic backgrounds, gave *P* values between 0.35 and 0.92. The rest condition consisted of 188 wild-type (wt) and 100 ko events, and the depolarized condition 228 wt and 60 ko events. The number of events per patch ranged from 32 to 99 for wt and from 10 to 50 for ko.

## Discussion

In this study, we made cell-attached C_m_ recordings on IHCs in a manner that left voltage-stimulated release operational and dependent on Ca_v_1.3 expression—hallmarks of normal IHC exocytosis. The results demonstrate that single fusion events form a size distribution that matches the range of SV diameters common to IHC active zones. This relationship is maintained under different experimental conditions, suggesting that release from IHCs is predominantly univesicular. The findings are consistent with numerous studies that measured C_m_ changes with the cell-attached method and reported that the amount of membrane added per fusion event correlated with the morphological size of secretory vesicles expressed in each of the cell types (13–15, 21, 24). Since this represents one of a few on-cell C_m_ studies to measure small vesicle fusion events, we start by comparing our findings to those studies and then discuss our results in the context of hair cell synaptic transmission.

The study by He et al. (13) first reported that individual C_m_ fusion events measured at the synaptic face of the calyx of Held nerve terminal yielded steps of ∼73 aF, close to what could be expected for the fusion of single SVs averaging 45 nm in diameter. They later showed that when the cells were depolarized by elevating extracellular KCl (50–100 mM) such that a greater proportion of large “compound” vesicles appeared in the electron micrographs, the outcome was a direct increase in the percentage of larger fusion events (16). This study points to a plasticity in vesicle biogenesis upstream of exocytosis. In a different study that focused on release from posterior pituitary nerve terminals, Klyachko and Jackson (15) showed that C_m_ fusion event amplitudes were bimodally distributed with peaks at 67 and 412 aF, which corresponds to the size of synaptic-like microvesicles and granules expressed in these cells (vesicle diameters of 48.5 and 125 nm, respectively). The fusion step size distribution remained bimodal at rest when event frequency was ∼0.1 Hz, and during high KCl stimulation when event frequency increased to ∼0.5 Hz. For comparison, we directly depolarized the patch of membrane relative to the ground electrode and witnessed an elevated event frequency (0.45 Hz), and the outcome was steps of ∼40 aF, closely corresponding to SVs with diameters of 36 nm. In total, these studies argue against MVR release being coordinated at the plasma membrane and instead suggest that the amplitudes of fusion events reflect the distribution of SV sizes.

There are potential drawbacks associated with on-cell C_m_ measurements. One concern is that when the rate of events is kept low so that individual events are well separated, not overlapping, this may preclude an MVR process. However, coordinated MVR does not rely on the random superposition of events and therefore absolute release frequency is not a prerequisite (3). What is more difficult to rule out is whether the on-cell configuration perturbs a mechanism that coordinates SV fusion. As highlighted above, the patches of membrane do exhibit functional behavior; therefore, we favor the view that the recordings reflect presynaptic physiology.

So, how can UVR account for the amplitude diversity of sEPSCs in IHCs? Previous studies using amperometry to measure the amount of transmitter released per fusion event have shown a strong correlation between quantal size and vesicle volume (35, 42–44). However, when making recordings from the postsynapse, it is difficult to know how much transmitter is released per EPSC since synaptic structure and receptor properties codetermine the response (for review, see refs. 41 and 45). Nonetheless, the amount of transmitter released per SV is thought to have the greatest influence on miniature EPSC (mEPSC) amplitude variance (16, 34, 40, 46). Bekkers et al. (34) put forth the argument that variance in SV volume directly impacts quantal variance and, in doing so, contributes to mEPSC variance. A detailed modeling study on the topic concluded that SV diameters with a CV = 0.2 would yield mEPSC amplitudes with a CV = 0.61 (see table 1 in ref. 36). After removing the largest 6% of the C_m_ fusion events measured here, the equivalent SV volumes have a CV > 0.6 (CV for equivalent diameter: 0.21), which exceeds the variance associated with K^+^ evoked EPSCs measured from 3-wk-old wild-type mice that were reported to consist mostly of monophasic EPSCs (>92% of the events) with a CV ∼0.33 (37). Hence, the variance in the size of fusing SVs is more than enough to account for variance in EPSCs measured from 3-wk-old mice.

It is known that IHC-SGN synaptic structure is refined during development. This is accompanied by more efficient secretion coupling (47), and the EPSCs change significantly through development. Earlier studies examined the influence of development on EPSCs in young rats [postnatal day (P)7 to P21] and found that with age the monophasic EPSCs became more frequent (going from 59 to ∼72%), developed faster waveform kinetics, and were shifted to larger amplitudes, which created an unusual skew toward small events (3, 10). Likewise, prehearing mice, P9 to P11, show significant variance in EPSC waveform: ∼60% monophasic and 40% multiphasic, and the amplitude distributions for either type of waveform are heavily skewed toward larger events (38), while 3-wk-old mice are almost entirely monophasic (37). It is worth noting that EPSCs measured from mature bullfrog auditory hair cells are exclusively monophasic (39). mEPSCs measured from calyx of Held are monophasic and they to show an age-dependent enhancement in mEPSC amplitude and an acceleration in waveform kinetics through development (48). These age-related changes are widely viewed as improving signaling efficiency (10, 49), but as to how this is achieved is less clear at the IHC-SGN synapse. In the context of a UVR process, the multiphasic events witnessed in young mice may reflect release through a dynamic fusion pore (9), or possibly other presynaptic and postsynaptic changes yet to be identified. However, within the set of monophasic EPSCs, the developmental changes match what has been described for partial receptor saturation under a UVR (see figure 8 in ref. 50), and a common feature associated with saturation is a reduction in variance and a skew toward smaller events (for review, see ref. 45). Partial saturation of IHC-SGN receptors could dampen amplitude variance and potentially explain why variance in SV volume exceeds the variability in EPSC amplitude.

Is there sufficient glutamate in a quantum to support signaling through a UVR process? Franks et al. (36) modeled different presynaptic and postsynaptic arrangements for a simple central synapse, and 2,000–3,000 molecules of glutamate released per SV was sufficient to yield realistic outcomes for UVR. A similar number of glutamate molecules was found to be adequate to model UVR at the IHC-SGN synapse (9). The latter study explored two separate AMPA receptor kinetic schemes, both of which were able to yield ∼120–240 pA EPSCs when the quantum equaled 3,000 molecules. This EPSC amplitude approximates what has been measured in 3-wk-old mice. Packing 3,000 glutamate molecules into a 37-nm diameter SV would yield an intravesicular concentration of ∼200 mM glutamate. Biochemical (51) and physiological (48) studies suggest that this amount of glutamate in an SV is reasonable, and other neurotransmitter systems are believed to concentrate transmitters in SVs to 100–300 mM (35, 52, 53).

Since a spontaneous EPSC can effectively trigger an SGN-AP (5), and if UVR prevails at this synapse as suggested here and earlier (9), then single SV fusion events should be sufficient to drive SGN spiking. To our knowledge, this level of efficiency, 1 SV to 1 AP, is unprecedented. Interestingly a single hippocampal mossy fiber-CA3 synaptic contact is capable of driving APs, similar to a IHC-afferent contact, yet the quantal content underlying the MF release remains unknown (54). It may seem odd that a ribbon-type AZ would have so many SVs, but only utilize them one at a time. However, a 1 SV to 1 AP ratio is maximally efficient, and this will help IHCs maintain high rates of continual release by mitigating the onset of presynaptic fatigue and reduce the metabolic burden of excess glutamate recycling.

## Methods

The animals were under the care of the Max Planck Institute for Biophysical Chemistry. The apical coil of cochlea from male and female mice 4–8 wk old were used. Mice were maintained on a C57BL6/J background with/without Ca_v_1.3 knockout (4, 32). Control mice were either wild type (^+/+^) or heterozygous (^+/−^) for the wild-type Ca_v_1.3 gene, and the knockouts were homozygous (^−/−^). For further details on electrophysiological methods, see *SI Appendix*, *Methods*.

## Supplementary Material

Supplementary File
