# Supplementary material for: Individual synaptic vesicles mediate stimulated exocytosis from cochlear inner hair cells
Source: Proc Natl Acad Sci U S A. 2018 Nov 21;115(50):12811–6. doi: 10.1073/pnas.1811814115 (PMC6294930; doi:10.1073/pnas.1811814115)
Supplement: Supplementary File [file pnas.1811814115.sapp.pdf]

PNAS
www.pnas.org

Supplementary Information for

Individual synaptic vesicles mediate stimulated exocytosis from cochlear inner hair cells

Authors: Chad Paul Grabner, and Tobias Moser

corresponding author: Chad Grabner
Email: chadgrabner@gmail.com

This PDF file includes:

Supplementary text
Figs. S1 to S3
References for SI reference citations

www.pnas.org/cgi/doi/10.1073/pnas.1811814115

Supplementary Information Text

Signal Processing. Evaluating the Real part of the Lock-in signal for signs of a fusion pore conductance in the context of signal smoothing. The influence low-pass filtering and smoothing have on the Real (Y1) and Imaginary (Y2) parts of the Lock-in signal are in principle the same. However, the potential information carried in the Real and Imaginary signals offers distinct insight into vesicle fusion, and therefore the consequences of signal smoothing are not necessarily the same. Before examining how signal processing shapes fusion related signals, we first give a demonstration of how the Real and Imaginary parts were assigned to conductance and capacitance signals, respectively.

Three segments from an individual on-cell recording episode are presented in Figure S1. In the first example, the Imaginary part showed an up-step without accompanying changes in the Real or membrane current (I_m) traces (Fig. S1A). In the second example, the Real part fluctuated along with the I_m (Fig. S1B), and the Imaginary part was steady throughout. Finally, at the end of the recording the system was calibrated by dialing in 2fF (peak-to-peak) into the fast capacitance compensation (C_{fast}) on the front panel of the EPC-7 amplifier. The adjustment to C_{fast} only changed the Imaginary part (Fig. S1C). These examples support the designation of the Imaginary part as C_m, and distinct from the Real part that represents an aggregate of conductance (G). The Real part includes membrane (G_m) and potentially pore (G_p) conductances. The example in

Fig. S1B is considered to represent a change in G_m, but not G_{pore}, because the fluctuation in G is not associated with any obvious change in the C_m that would represent membrane fusion (1, 2).

The transient nature of fusion pores makes them prone to being lost with smoothing (3-5). For this reason, the G and C_m signals were inspected at full bandwidth (without smoothing) and after signal smoothing (32k-point Binomial smooth). Signal processing did not reveal changes in conductance at the onset of fusion steps that could be interpreted as a fusion pore signal, nor did fusion events display a steady G_{pore}. For example, see Figure 1A and 1B to compare traces before and after smoothing. The ΔC_m events in these examples are not accompanied by a ΔG that is suggestive of a pore conductance.

The fusion events reported here averaged 40 aF, which are almost 50 % smaller than all former studies using the on-cell method. The magnitude of the G_{pore} signal is determined by the size of the ΔC_m step (1, 6, 7), and it has been predicted that to measure a G_{pore} signal from a 80 aF fusion event, an rms-noise of 1 aF is needed (1). To measure a fusion pore from our small SVs would require an rms-noise < 1 aF, which is a noise level less than what we or others have achieved. Actual studies examining small vesicle fusion steps averaging ~70 aF, have found that full-fusion events (simple up-steps in C_m) lacked a detectable G_{pore} (4) or only 1.4 % of the full-fusion events had a G_{pore} signal (5) (nearly 94 % of our events were full-fusion). These previous studies confirm that pore conductance is difficult to measure from small vesicles.

Estimating fusion event amplitudes and rise times after smoothing. C_m and G_m Lock-in traces were low-pass filtered with a time constant of 100 μs prior to sampling at 50

kHz, and then smoothed offline with a 32,000-point Binomial smooth (see Methods for details). To estimate the influence of smoothing on event rise time and amplitude, two signals were evaluated. First, a defined signal: a voltage step, and second a C_m trace with a fusion event. The voltage step was taken from the actual command voltage used in experiments. The voltage signal was sampled in parallel at the same rate as the G and C_m traces, and it contained the 58.5 kHz sine-wave used in the Lock-in analysis. A 0.1k-point Binomial smooth removed most of the sine wave and yielded a rise time 20-80 % (rt20-80) = 0.2 ms (Fig. S2A). Further smoothing with 2k- and 32k-points increased the rt20-80 to 1 and 3.4 ms, respectively (Fig. S2A). After the steps settled they reached a similar final step amplitude regardless of the number of points used for smoothing (Fig. S2A).

Figure S2B examines the influence of smoothing on a C_m trace with a fusion event. The obvious benefit of smoothing is a reduction in C_m rms-noise (Fig. S2C). The trace at full-bandwidth contains ~30 aF rms-noise (Fig. S2B-D, black trace), and after smoothing the signal has less than 5 aF rms-noise (Fig. S2B, red and green traces: 2k and 32k points). There was no change in fusion event amplitude with progressively more smoothing, amplitudes: 54, 54 and 56 aF following 0.1k, 2k and 32K point Binomial smoothes, respectively (Fig. S2C,D, white traces are Logistic function fits). In contrast to the ideal voltage step which showed attenuation in rising time after smoothing, the rt20-80 measurements of the actual fusion events were similar across the different smoothing conditions: 5.2, 5.2 and 6.2 ms (0.1, 2 and 32 kHz pt-Binomial smoothes, respectively; Fig. S2C,D). One possible explanation for the result is that the true rise time is slower

than the limit imposed by smoothing, and an alternative scenario is that noise in the C_m signal interferes with analysis of the rising phase.

To simulate the influence baseline noise has on event rise time, a hybrid trace was constructed by adding a series of small voltage steps (taken from the voltage traces as described above; Fig. S2E) to segments of C_m baseline that were processed with 32k-point Binomial smoothing (Fig. S2F,G). The results show that baseline noise lengthened the average hybrid steps rise by 2-fold. The original steps had rt20-80 values of 4.9 ± 0.2 ms versus the hybrid steps: 10.8 ± 3.4 ms (mean ± s.e.). The difference was not statistically significant (unpaired Two-Sample t-Test p: 0.24; n=16 steps in original and hybrid steps), because the hybrid steps had too variable. The hybrid steps had rt20-80 values that ranged from 2.7 to 45 ms (CV = 1.3), and voltage steps without added noise had rt20-80 values of 4.4 to 5.8 ms (CV = 0.1). The step amplitudes were similar for the voltages step with and without added noise, 50 ± 2 aF and 47 ± 0.2 aF, respectively ($p > 0.2$) (the original step was set to 47 aF, Fig. S2E).

The rt20-80 was measured for IHC. Though the signals were overly attenuated, the values are presented as a check on the analysis. When fusion step height was measured, as presented in Figure 4 and in Table 1, the rt20-80 for these events had the following median values (mean ± s.e.), wild-type: 8.7 ± 0.8 ms (n: 7 patches) and ko: 9.1 ± 0.9 ms (n: 5 patches). There was no significant difference between the two groups (p: 0.75).

The analysis shows that estimation of rt20-80 is susceptible to interference from baseline fluctuations (noise), which can attenuate the true rise time by > 6 msec. Thus, using C_m as a indicator for vesicle fusion flickers or pore expansion is unlikely to offer

insight into the sub-millisecond transitions in transmitter release (8-10) that have been proposed to generate the monophasic and multiphasic transitions in the EPSC signals (11, for review see: 12).

Kiss-and-run events have been described by others as C_m steps with similar rising and falling phase kinetics (4, 5, 13). Such events were not apparent in our recordings. Interestingly, a fraction of the fusion events were succeeded by a relatively slower decaying C_m that may represent a membrane retrieval process. Examples of individual C_m up-steps followed by C_m decays are presented in Figure S3. Some of the events showed a decay in C_m that was not possible to discern from a drift towards baseline (Fig. S3A), while others showed a relatively faster decay phase that could be quantified (Fig. S3B, C). Inspection of 3 wild type cells with low noise levels (~4 aF rms-noise) yielded 16 up-steps followed by a C_m decay transition (8 % of total up-steps, 16/201). The event half-width (HW) was measured to summarize event duration (Fig. S3C), and yielded a mean HW = 471 ± 67 ms. This estimate of duration falls within the range of kiss-and-run event durations that have been reported for SVs and granules: 270 ms (5) and 560 ms (4), respectively. Next, the up- and down-step amplitudes were averaged and found to have similar mean amplitudes: 41 ± 4 aF and 40 ± 4 aF (n=16), respectively, and when the ratio of pairs of up and down step amplitudes were computed, the mean ratio was close to unity: 1.01 ± 0.09. In contrast, the kinetics of event rise and fall were very distinct. The average fall time constant was 66.3 ± 18.2 ms and universally slower than the average rise time constant: 3.3 ± 0.7 ms (p: 0.002; n = 16).

These transient steps were not associated with a change in G that could be argued to represent a fusion pore conductance. In total, the relatively slow decay in C_m and the

lack of a pore conductance fit the description of 'pseudo-flicker' events. Such events have previously been described in a study that made on-cell C_m recordings from human neutrophils (14), and therein is ample discussion on what these events represent.

Methods

Electrophysiological recordings. The extracellular recording solution had the following composition (mM): 3 KCl, 145 NaCl, 2 $CaCl_2$, 1.5 $MgCl_2$, 10 HEPES and 10 Glucose; pH= 7.3 and osmolarity = 290 mOsm. Dissections were made in the same extracellular media except the $CaCl_2$ was lowered to 0.5 mM. After dissection, the tissue was continually perfused in 2 mM Ca^{2+} extracellular solution until the recording started. Minimal cleaning of the IHC's basolateral surface was made by separating supporting cells and boutons from the IHCs with moderate positive pressure on the cleaning pipette (10 min preparation time). Recording pipettes were pulled from thick wall borosilicate glass, slightly fire polished and then carefully coated with Sylgard. Electrodes were filled with Ringer's solution with 20 mM TEA-Cl and 3 mM CsCl in place of 20 mM NaCl, pH 7.3.

Before starting recordings, the surface of the bath solution was swept free of buoyant fatty residues. A clean recording electrode was advanced across the bath with positive pressure applied to the pipette solution. The Sylgard coated region of the electrode was fully immersed, 'wetted', and then the height of the bath solution on the Sylgard contact zone was lowered to relieve solution surface tension on the electrode. This approach prevented solution from creeping up the electrode which would otherwise tend to result in undulations in the C_m signal (see Chapter 17 in Sakmann and Neher (15)). Finally, the bath solution was lowered to the minimal amount permitted, and then the recording electrode was sealed onto the basolateral end, and modiolar side of the IHC. Rapid seal formation was achieved without drawing a tongue of membrane into the pipette. Only one patch was made per cell, and typically one cell per cochlea. Variability in event frequency was witnessed between patches, and this is summarized in Figure 3.

Patches without events were not observed, though some patches had few events. A few wild type cells were too active to analyze.

Hardware. An EPC-7 amplifier (HEKA; Lambrecht, Pfalz; Germany) was configured with a Lock-in amplifier model SR830 from Stanford Research Systems (Sunnyvale, CA; USA) as described previously (16) to monitor changes in membrane electrical impedance. Briefly, a 58.5 kHz sine wave with a V_{rms} between 130 to 200 mV was fed into the EPC-7 V_{input} with the input time constant set to 0.2 µs. The EPC-7 gain was set to 50 mV/pA (using the 100 GΩ feedback resistor), and the signal output was sent at full-bandwidth to the Lock-In amplifier. The Y1 (Real, G) and Y2 (Imaginary, C_m) outputs were filtered to a 100 µs time constant with a 24 dB roll-off (SRS830 internal filter), and sampled at an interval of 20 µs using a connector box and A/D card (SCB-68A and NI PCIe-6321, respectively, from National Instruments, Austin, TX; USA) that was run by software from NI and Igor NIDAQ Tools (Wavemetrics, Portland, OR; USA). The unfiltered membrane current was passed through a 5 kHz Low-pass Bessel filter (Frequency Devices, Haverhill, MA, USA) and then collected along with the $V_{applied}$ ($V_{monitor}$ from EPC-7) as well as with Y1 and Y2. Recording epochs were collected over 120 to 300 sec, ~1 to 4 epochs per patch. A C_m calibration procedure was made per epoch, which consisted of ±1 fF steps introduced via a well calibrated C_{fast} circuitry on the front panel of the EPC-7 (see Fig. S1C). The Y1 (Real, G) and Y2 (Imaginary, C_m) Lock-in signal outputs were 90° out of phase as evidenced by 1) the exclusive sensitivity of Y2 to a charged body (hand) approaching the open headstage input, and 2) adjustments to C_{fast} on the EPC-7 only altered Y2 (C_m; see Fig. S1C). Further phase adjustment to actual C_m and G traces from patch recordings were made off-line using software provided

by Manfred Lindau's group, which is described elsewhere and available online (16). Most recordings required a phase adjustment of ± 5° from the original recording phase (0°) (as in Fig. S1). Once the C_m signal was phase adjusted and deemed not to project into the G_m signal, fluctuations in membrane current (I_m) (which appeared to be K^+ channels since E_{rev} was ~ E_K) created a corresponding change in G_m without altering C_m (as in Fig. S1).

Data analysis. After calibration and phase adjustment, G and C_m traces (16) were low-pass filtered offline with a Binomial filter routine (Igor Pro software, Wavemetrics; Portland, OR; USA) to yield a rise time 20-80 % (rt20-80 %) of ~4 ms. Filtered traces were downsampled from 50 to 10 kHz and then imported into the event detection program MiniAnalysis (SynaptoSoft, Decatur, GA; USA). The following parameters were entered into the analysis program: event threshold: 20 aF (which was 5-8 x rms-noise), time for a local maximum: 100 ms, time before a peak for baseline: 300 ms, period to average a baseline: 100 ms, and an event area threshold: 1 fF. In segments where the steps were crowded, the local max and time before peak for baseline were reduced to 50 ms and 100 ms, respectively, which created a ~100 ms baseline for the next step in the sequence. In most instances, especially during low event frequency, the capacitance steps made a smooth uninterrupted ascent with a rt20-80 % in under 10 ms (see Fig. S2). Overlapping events were not analyzed since a baseline could not be established. After the events were detected, they were aligned to their rising phase, baseline subtracted, and then the rising phase was fit with a sigmoid function ('Logistics 5' function; OriginLab; Northampton, Mass., USA). The following parameters were obtained from the sigmoid fits: 1) rt20-80 % (ms) and 2) step amplitude (aF): the

difference between the pre- and post-step levels. OriginLab was used for statistical comparisons of unpaired samples, using the Two-Sample t-Test, and equal variance was not assumed (Welch correction).

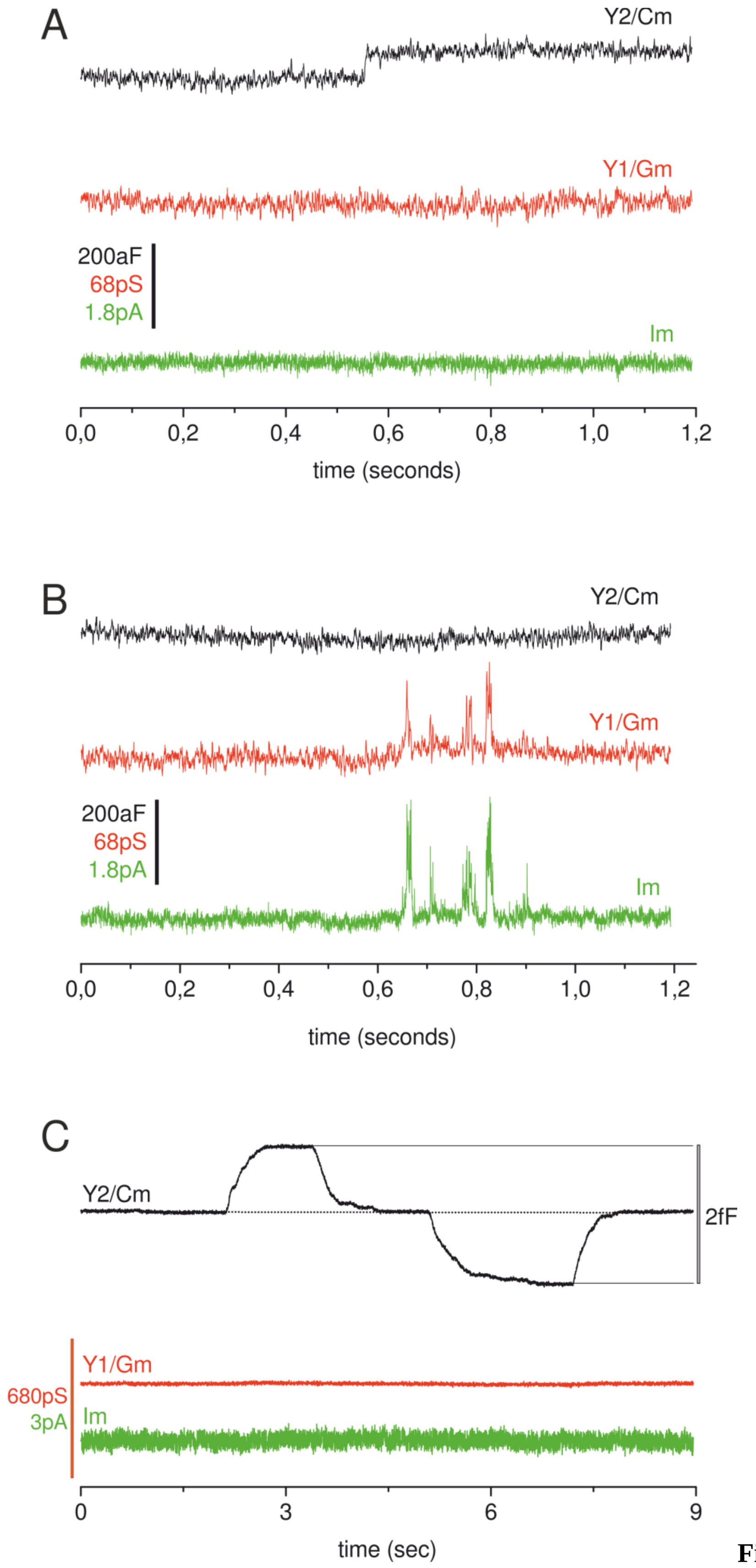

A
Y2/Cm
Y1/Gm
200aF
68pS
1.8pA
Im
0,0
0,2
0,4
0,6
0,8
1,0
1,2
time (seconds)
B
Y2/Cm
Y1/Gm
200aF
68pS
1.8pA
Im
0,0
0,2
0,4
0,6
0,8
1,0
1,2
time (seconds)
C
Y2/Cm
2fF
Y1/Gm
680pS
3pA
Im
0
3
6
9
time (sec)

Figure S1.

Figure S1. Examples of Real (Y1) and Imaginary (Y2) components of the Lock-in signal in relation to the membrane current (I_m). **A**, This segment of the recording shows a step in the Imaginary signal without concurrent changes in Real or I_m signals; therefore, Y2 is deemed to represent membrane capacitance (C_m). **B**, Here a fluctuation in membrane current was reflected in Y1, without any influence on Y2. Such changes in Y1 represent a change in membrane conductance (G_m), or other changes in conductance like seal resistance. **C**, Upper panel shows the calibration of Y2 which consisted of dialing 2fF into the amplifiers C_{fast} compensation circuit (see Methods). Importantly, the adjustment in Y2 did not influence Y1, indicating the separation of Y1 and Y2. Traces in A-C are from a single 4 minute recording episode, and the Lock-in traces were phase adjusted offline using a phase angle of -4^0 .

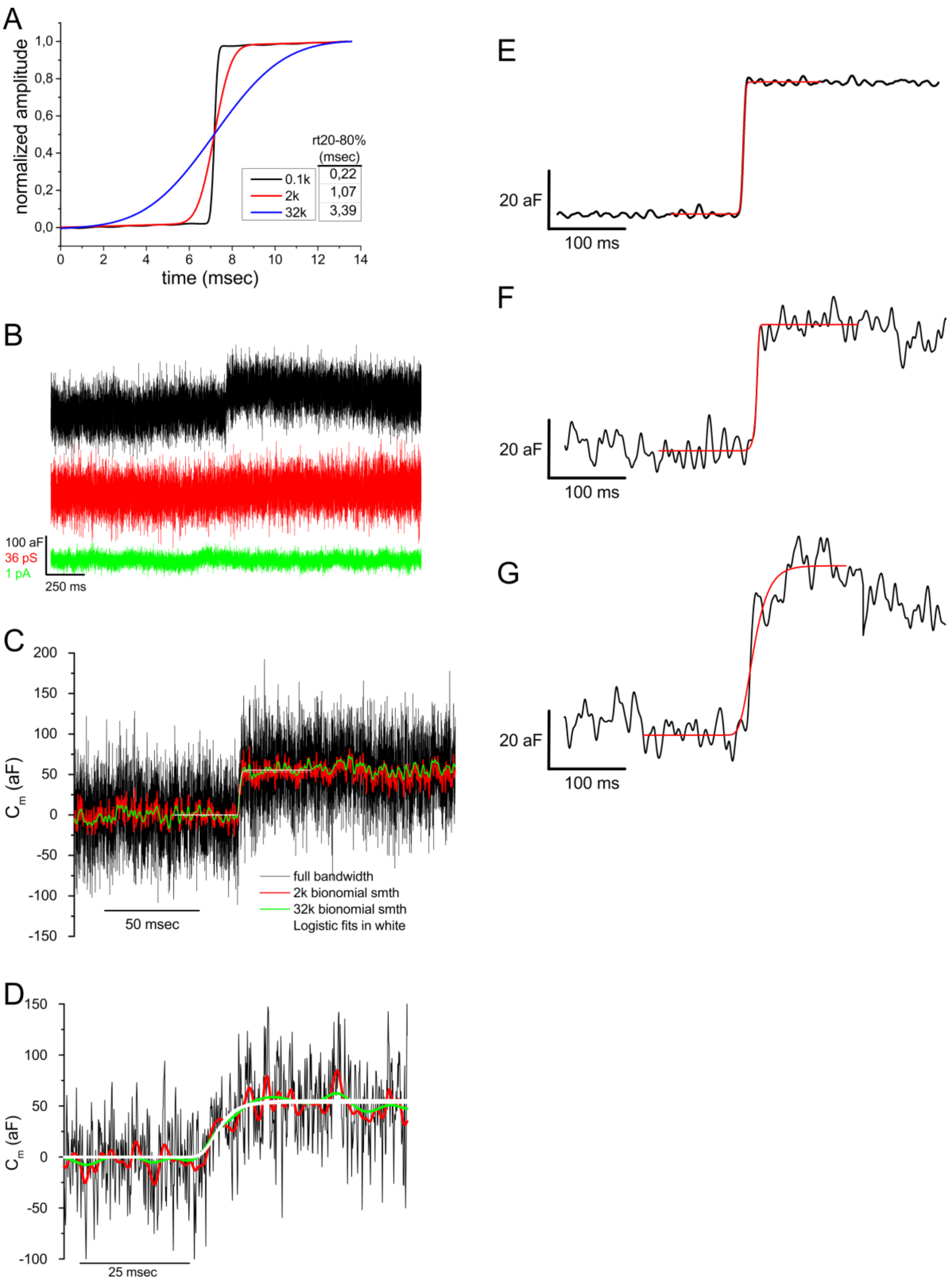

Figure S2. Influence of low-pass filtering on fusion events. **A**, Voltage step smoothed with a Binomial routine, using the following number of points: 0.1k, 2k and 32k-points. **B**, Membrane current (green), C_m (black), and G (red) traces presented without smoothing. **C**, C_m trace from (**B**) with progressive smoothing and Logistic fits made to each of the three C_m traces. **D**, Zoomed

view of (**C**). **E**, Voltage step smoothed with 32k-pt Binomial smooth and its amplitude arbitrarily scaled to 47 aF. **F** and **G**, Hybrid steps made from voltage step in (**F**) plus baseline noise. The Logistic function was fit to steps in E-G.

.

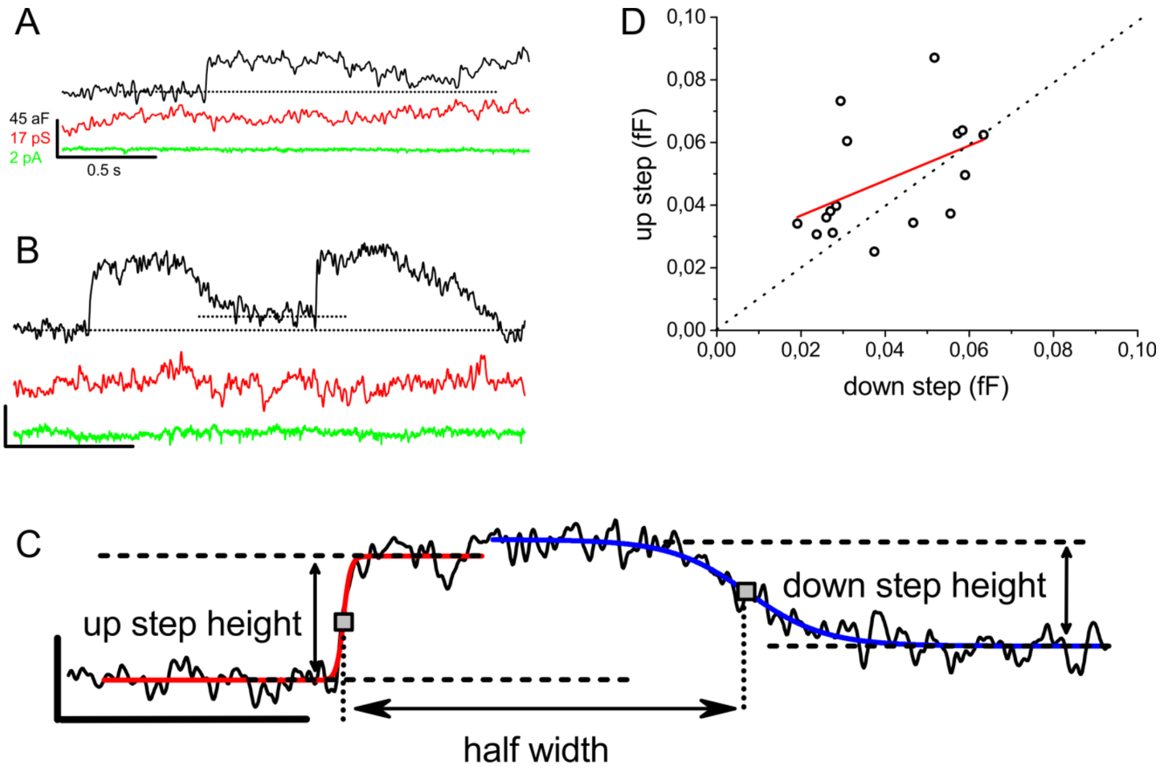

Figure S3. Pseudo-flicker fusion events have a rapid rise and slow decay. **A** and **B**, the up-steps in C_m (black trace) of a wild-type IHC appear in the absence of a correlated change in G (red trace) or I_m (green trace). **A**, The small fusion event is followed by a slow decay towards baseline, while in (**B**) the two steps show relatively faster and more obvious decays towards baseline, and are referred to here as pseudo-flicker events. **C**, Boltzmann fits to the rising (red line fit) and falling phase (blue line fit) were made to the first event in (**B**) to illustrate how event amplitude, and kinetics were measured. Half-width determination for these asymmetrically shaped events were measured as the time between the EC_{50} rise and EC_{50} fall (EC_{50} is designated with grey boxes). **D**, Plot of up- versus down-step amplitude for each fusion event (linear fit to data in red). The points are scattered around unity (dashed line). Scale bars are the same for (**A-C**).

SI Appendix References

1. Debus K & Lindau M (2000) Resolution of patch capacitance recordings and of fusion pore conductances in small vesicles. *Biophysical journal* 78(6):2983-2997.
2. Albillos A, *et al.* (1997) The exocytotic event in chromaffin cells revealed by patch amperometry. *Nature* 389(6650):509-512.
3. Henkel AW, Meiri H, Horstmann H, Lindau M, & Almers W (2000) Rhythmic opening and closing of vesicles during constitutive exo- and endocytosis in chromaffin cells. *The EMBO journal* 19(1):84-93.
4. Klyachko VA & Jackson MB (2002) Capacitance steps and fusion pores of small and large-dense-core vesicles in nerve terminals. *Nature* 418(6893):89-92.
5. He L, Wu XS, Mohan R, & Wu LG (2006) Two modes of fusion pore opening revealed by cell-attached recordings at a synapse. *Nature* 444(7115):102-105.
6. Spruce AE, Breckenridge LJ, Lee AK, & Almers W (1990) Properties of the fusion pore that forms during exocytosis of a mast cell secretory vesicle. *Neuron* 4(5):643-654.
7. Jorgacevski J, *et al.* (2010) Fusion pore stability of peptidergic vesicles. *Molecular membrane biology* 27(2-3):65-80.
8. Grabner CP & Zenisek D (2013) Amperometric resolution of a prespike stammer and evoked phases of fast release from retinal bipolar cells. *The Journal of neuroscience : the official journal of the Society for Neuroscience* 33(19):8144-8158.
9. Stiles JR, Van Helden D, Bartol TM, Jr., Salpeter EE, & Salpeter MM (1996) Miniature endplate current rise times less than 100 microseconds from improved dual recordings can be modeled with passive acetylcholine diffusion from a synaptic vesicle. *Proceedings of the National Academy of Sciences of the United States of America* 93(12):5747-5752.
10. Staal RG, Mosharov EV, & Sulzer D (2004) Dopamine neurons release transmitter via a flickering fusion pore. *Nature neuroscience* 7(4):341-346.
11. Chapochnikov NM, *et al.* (2014) Uniquantal release through a dynamic fusion pore is a candidate mechanism of hair cell exocytosis. *Neuron* 83(6):1389-1403.
12. Lisman JE, Raghavachari S, & Tsien RW (2007) The sequence of events that underlie quantal transmission at central glutamatergic synapses. *Nature reviews. Neuroscience* 8(8):597-609.
13. Ales E, *et al.* (1999) High calcium concentrations shift the mode of exocytosis to the kiss-and-run mechanism. *Nature cell biology* 1(1):40-44.
14. Lollike K, Borregaard N, & Lindau M (1998) Capacitance flickers and pseudoflickers of small granules, measured in the cell-attached configuration. *Biophysical journal* 75(1):53-59.
15. Sakmann B & Neher E (2009) *Single-channel recording* (Springer, New York, NY) 2nd Ed pp xxii, 700 p.
16. Dernick G, Gong LW, Tabares L, Alvarez de Toledo G, & Lindau M (2005) Patch amperometry: high-resolution measurements of single-vesicle fusion and release. *Nature methods* 2(9):699-708.
